# In Vivo Identification of Skin Photodamage Induced by Fractional CO_2_ and Picosecond Nd:YAG Lasers with Optical Coherence Tomography

**DOI:** 10.3390/diagnostics12040822

**Published:** 2022-03-27

**Authors:** Chau Yee Ng, Tai-Ang Wang, Hsiang-Chieh Lee, Bo-Huei Huang, Meng-Tsan Tsai

**Affiliations:** 1 Department of Dermatology, Chang Gung Memorial Hospital, Linkou Branch, Taoyuan 333, Taiwan; mdcharlene@gmail.com; 2 Graduate Institute of Clinical Medical Sciences, Chang Gung University, Taoyuan 333, Taiwan; 3 Vitiligo Clinic and Research Center, Chang Gung Memorial Hospital, Linkou Branch, Taoyuan 333, Taiwan; 4 Graduate Institute of Photonics and Optoelectronics, National Taiwan University, Taipei 106, Taiwan; d10941014@ntu.edu.tw (T.-A.W.); hclee2@ntu.edu.tw (H.-C.L.); 5 Department of Electrical Engineering, National Taiwan University, Taipei 106, Taiwan; 6 Department of Electrical Engineering, Chang Gung University, Taipei 333, Taiwan; akohuang0408@gmail.com; 7 Department of Biomedical Engineering, Chang Gung University, Taipei 333, Taiwan; 8 Department of Neurosurgery, Chang Gung Memorial Hospital, Linkou Branch, Taoyuan 333, Taiwan

**Keywords:** photodamage, therapy, optical coherence tomography, skin, diagnosis

## Abstract

Fractional laser treatment is commonly used for dermatological applications, enabling effective induction of collagen regeneration and significantly reducing recovery time. However, it is challenging to observe laser-induced photodamage beneath the tissue surface in vivo, making the non-invasive evaluation of treatment outcomes difficult. For in vivo real-time study of the photodamage induced by fractional pulsed CO_2_ and Nd:YAG lasers commonly utilized for clinical therapy, a portable spectral-domain optical coherence tomography (SD-OCT) system was implemented for clinical studies. The photodamage caused by two lasers, including photothermal and photoacoustic effects, was investigated using OCT, together with the correlation between photodamage and exposure energy. Additionally, to investigate the change in the optical properties of tissue due to photodamage, the attenuation coefficients and damaged areas of normal skin and laser-treated skin were estimated for comparison. Finally, the recovery of the exposed skin with both lasers was also compared using OCT. The results show that OCT can be a potential solution for in vivo investigation of laser-induced tissue damage and quantitative evaluation.

## 1. Introduction

Photon energy can be easily absorbed by melanin and further transferred to the thermal effect. Such photon energy can be transformed into photothermolysis for therapeutic applications [1,2]. Additionally, photothermolysis is commonly applied to skin resurfacing, which can remove skin tissue to induce regeneration of skin cells, making skin tighter and smooth [3]. However, conventional laser skin resurfacing causes severe side effects and requires a lengthy recovery. In contrast, fractional ablative photothermolysis, such as fractional CO_2_ laser, effectively reduces the tissue recovery period through fractional mode, sparing tissue between the lasers to enhance regeneration [4]. Fractional lasers can create microthermal ablation zones (MAZs) beneath the skin surface, causing striped thermal damage from the epidermis to the dermis layer surrounded by undamaged tissue. Typically, the heat produced by the laser is applied to the skin in the order of a millisecond. Previous reports also indicate that such induced MAZs can effectively reduce tissue resurfacing recovery time compared to conventional laser resurfacing [5,6,7]. Since ablative lasers cause thermal damage to tissue, temperature monitoring in tissue can be an excellent indicator to investigate the induced thermal damage. Sobol et al. proposed a theoretical model to evaluate the temperature field of cartilage induced by laser radiation [8].

Compared with fractional laser treatment, fractional picosecond lasers have attracted a lot of attention for dermatological applications because of their much shorter recovery period with less post-inflammatory hyperpigmentation. Picosecond lasers can cause laser-induced optical breakdown (LIOB) via multiphoton ionization, simultaneously resulting in high pressure and high temperature in the skin [9,10]. Such high pressure and temperature are usually combined with plasma expansion to further induce vacuolization beneath the skin surface, decomposing tiny pigments [11,12]. Therefore, the pressure wave can propagate from a shallow skin depth into the dermis, further triggering the tissue recovery. Recently, picosecond lasers have been used to treat pigmentation, acne scars, wrinkles, photodamaged skin, and tattoo removal by producing uniform vacuoles to ablate skin pigments with minimal damage to the surrounding tissue [13,14,15]. The fractional mode in picosecond lasers has been adopted in skin collagen regeneration, skin resurfacing, pore minimization, acne scars, and wrinkles.

Although various lasers have been proposed for dermatological applications, it is challenging to observe photodamage and repair progress induced by laser treatment in vivo. Additionally, as previously mentioned, picosecond lasers create vacuoles that can cause tissue fragmentation and regeneration. However, the diameters of tiny vacuoles are approximately tens of micrometers and are distributed over the epidermis and the shallow dermis layer [16]. Moreover, the existence and size of vacuoles are unpredictable. The induced photodamage, including photothermal and photoacoustic effects, is related to the optical properties of the skin, laser wavelength, incident photon energy, spot size of the treatment laser, focusing depth, and melanin [11,17]. The photodamage in clinical applications is unpredictable and varies between individuals. Thus, a monitoring tool for real-time non-invasive and in vivo observation of laser treatment outcomes is required to improve the treatment accuracy and prevent additional adverse events. In previous reports, various imaging techniques were proposed to investigate laser treatment outcomes or guide laser treatment. Although histology is commonly used to study laser treatment outcomes, these processes are invasive, time-consuming, and complicated [18]. Previous reports also demonstrated that reflected confocal microscopy or confocal microscopy could help visualize the induced photodamage on the skin due to high imaging resolution [19,20]. However, the imaging depth of confocal microscopy was limited to 200 μm, making investigating the deeper structures difficult. Estrada et al. proposed a faster scanning biomicroscopy to simultaneously provide optoacoustic and pulse-echo ultrasound imaging to study the laser-induced damage on cartilage and sclera [21].

Moreover, Balu et al. proposed multiphoton microscopy for the in vivo study of LIOBs in human skin and characterization of the specific skin response [22]. However, the penetration depth is limited because a high-magnification objective is employed in microscopic systems, and the imaging sensitivity may not be sufficient to probe deeper skin structures. In contrast, optical coherence tomography (OCT) can provide the characteristics of non-invasive, in vivo, high-speed, label-free, and 3D imaging, enabling the investigation of photodamage from various view angles [23,24,25]. Owing to the development of Fourier-domain OCT, including spectral-domain OCT (SD-OCT) and swept-source OCT (SS-OCT), the imaging speed has been significantly improved to minimize motion artifacts during clinical scanning [26,27]. Furthermore, the design of handheld probes, such as galvanometer-based or single fiber-based configuration, facilitates in vivo scanning of arbitrary human skin locations [28,29,30,31]. In the previous study, OCT was utilized for ex vivo and in vivo monitoring of laser treatment outcomes by using the ablative fractional laser for the assistance of drug delivery [32].

In this study, a portable OCT device was used to investigate the photodamage at various depths beneath the skin non-invasively. The optical properties of the biological tissue including absorption and scattering, and the attenuation coefficients of the skin before and after laser exposure, were evaluated. The induced tissue damage area was quantitatively assessed. Finally, tissue recovery was also investigated using OCT.

## 2. System Setup and Methods

### 2.1. OCT System Setup

**Figure 1:**
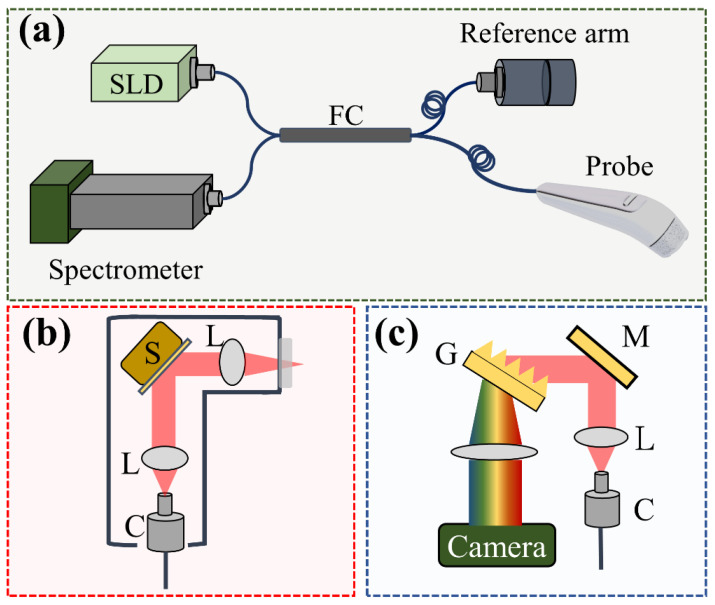
(**a**) Schematic diagram of the SD-OCT system for skin scanning, (**b**) design of the handheld probe with a MEMs scanner, and (**c**) optical setup of the spectrometer. S: MEMs-based scanner, L: lens, C: collimator, G: transmission grating, M: mirror.

In this study, a portable SD-OCT system (Optical Skin Viewer, OPXION Technology Inc., New Taipei City, Taiwan) was implemented to scan the skin. Figure 1 shows the setup of the SD-OCT system. A super-luminescent diode with a center wavelength of 840 nm was used as the light source. The full width at half-maximum of the light source was approximately 50 nm, corresponding to an axial resolution of 7 μm in the air. The light source was connected to a fiber coupler with a coupling ratio of 50/50, and the light beam from the light source was divided into reference and sample arms. A handheld probe was designed for scanning arbitrary skin locations in the sample arm, and a MEMs-based scanner was used to provide the lateral and raster scans, as shown in Figure 1b. Additionally, a focus lens with a focal length of 18 mm was inserted at the output end of the probe to focus the light beam on the skin. The corresponding lateral resolution was approximately 10 μm. The reference arm comprised a fiber collimator, a reflective mirror, and a focusing lens, the same as that used in the probe. Finally, the reflected/backscattered light from both arms was received by the spectrometer. The spectrometer was based on the composition of a transmission grating, lenses, and a line-scan camera. The line-scan camera’s line rate was set to 50 kHz, corresponding to a frame rate of 50 frames/s. Finally, the interference signal was detected by the spectrometer and resampled for wavelength calibration. The maximum scanning area for each volumetric scan can reach 5 × 5 mm^2^, which requires 10 s to acquire the 3D dataset and image rendering.

### 2.2. Lasers for Treatment

Given that the induced photodamage is related to treatment parameters such as the laser energy, spot size, and laser wavelength, two ablative lasers were used for comparison, namely a fractional CO_2_ laser and a fractional Nd:YAG laser. The fractional CO_2_ laser (UltraPulse ActiveFXTM, Lumenis, San Jose, CA, USA) is mainly used for skin resurfacing in clinical applications. The center wavelength of this fractional CO_2_ laser is 10,600 nm, and the pulse duration was set to 2 ms with a repetition rate of 300 Hz. Three energy levels (10, 20, and 30 mJ/microbeam) were applied to investigate the relationship between photodamage and exposure energy. The exposure area for each treatment was set to cover an area of 6 × 6 mm. In contrast, a fractional Nd:YAG laser (PicoWay, Candela, Marlborough, MA, USA) with a pulse width of 450 picoseconds was used to study the photodamage induced by picosecond lasers. Three output wavelengths, namely 532, 785, and 1064 nm, can be obtained from this picosecond laser. The laser repetition rate was set to 10 Hz, and the maximum exposure area for each treatment covered an area of 6 × 6 mm. In this study, a laser output of 1064 nm was used. Diffractive optical elements (DOEs) were implemented to produce multiple microbeams and to achieve a uniform distribution of laser beams on the skin. However, these DOEs cause energy loss and limit the maximal exposure energy per microbeam. The maximum exposure energy of the used picosecond laser can reach 3 mJ/microbeam. In this study, three energy levels, namely 1.3, 1.9, and 2.5 mJ/microbeam, were applied. The applied exposure energies of the fractional Nd:YAG laser were much lower than those of the fractional CO_2_ laser. Lower exposure energy of the fractional CO_2_ laser (less than 5 mJ/microbeam) is challenging to induce tissue ablation. Additionally, exposure to a higher energy level of the fractional Nd:YAG laser may cause photodamage on the skin surface (described in the Discussion Section). Therefore, the applied exposure energies of two lasers are different in this study. The repetition rates of fractional CO_2_ and Nd:YAG lasers were 300 and 10 Hz, respectively. The maximum exposure area for each treatment covered an area of 6 × 6 mm. The total exposure time in our experiments was less than 2 s.

### 2.3. Experimental Method

In this study, five healthy volunteers were involved, including two females and three males. The left and right forearm skin of each volunteer were chosen for laser treatment and OCT scanning. Before laser exposure, the selected skin region was scanned with OCT, and the same skin region was scanned again after laser treatment. The same skin region was repeatedly scanned every 24 h for follow-up observation after laser treatment. Each volunteer received laser treatments of two lasers on different skin regions. The experimental protocol of this study was approved by the Chang Gung Medical Foundation Institutional Review Board (IRB: 202000855A3C501). All experiments were performed with written informed consent according to the regulations and guidelines. Before laser treatment, an anesthetic ointment was applied to the exposed area to reduce the pain resulting from photodamage.

## 3. Results

### 3.1. Photodamage Induced by Fractional CO_2_ Laser

**Figure 2:**
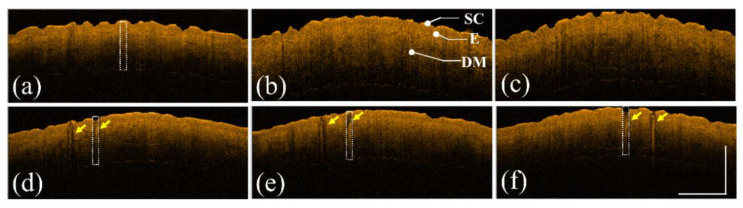
(**a**–**c**) 2D OCT images of healthy forearm skin of a 30-year-old female obtained before laser exposure. (**d**–**f**) OCT images corresponding to (**a**–**c**) after exposure to the fractional CO_2_ laser with energies of 10, 20, and 30 mJ/microbeam, respectively. The yellow arrows indicate the induced photodamage, and the scale bar represents 1 mm in length. SC: stratum corneum, E: epidermis, and DM: dermis.

First, the forearm skin of a 30-year-old female volunteer was exposed to a fractional CO_2_ laser. Three skin regions were exposed to different energy levels, including 10, 20, and 30 mJ/microbeam. Numbing cream was applied to the treated region before receiving laser treatment. Additionally, the same skin areas were repeatedly scanned using OCT before and after laser exposure. Figure 2a–c show the 2D OCT images of the healthy forearm skin obtained before laser exposure, and Figure 2d–f show the corresponding OCT images obtained from the exact skin locations in Figure 2a–c after laser exposures of 10, 20, and 30 mJ/microbeam, respectively. Different tissue layers, including the stratum corneum, epidermis, and dermis, can be identified in the OCT images, as indicated in Figure 2b. From the OCT results, it can be noted that the backscattered intensity of the exposed tissue became stronger than that of unexposed tissue. Moreover, because photon energy emitted from CO_2_ lasers can cause vertical photodamage from the skin surface into the dermis layer, the induced vertical damage is in the presence of a stripe structure, as shown in Figure 2.

**Figure 3:**
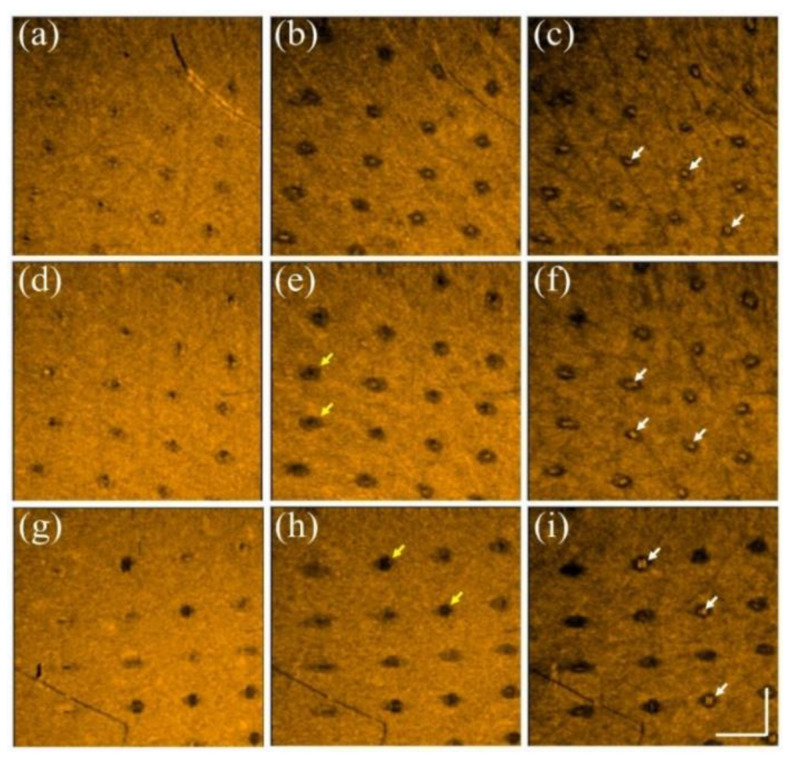
En face images of skin after exposure to the fractional CO_2_ laser with an exposure energy of (**a**–**c**) 10 mJ, (**d**–**f**) 20 mJ, and (**g**–**i**) 30 mJ/microbeam, obtained at depths of 200, 400, and 600 μm, respectively. The yellow and white arrows indicate the induced photodamage, and the scale bar represents 1 mm in length.

Additionally, to further investigate the photodamage at various skin depths, the corresponding en face images were extracted from the 3D OCT dataset, as shown in Figure 3. Figure 3a–c represent the en face images of the skin after laser exposure with an exposure energy of 10 mJ/microbeam obtained at depths of 200, 400, and 600 μm, respectively. Figure 3d–i show the en face images at depths of 200, 400, and 600 μm with exposure energies of 20 and 30 mJ/microbeam, respectively. The yellow arrows indicate the induced MAZs at shallower depths. The photon energy ablated the tissue in the center of the MAZ in the presence of a much lower backscattered intensity. Additionally, the white arrows indicate the MAZs at a deeper depth of 600 μm and show stronger backscattered intensity in the center of the MAZ. In contrast, because the tissue in the deeper layer was heated instead of undergoing tissue ablation, the backscattered intensity of the heated tissue became stronger, as indicated by the white arrows.

### 3.2. Photodamage Induced by Fractional Nd:YAG Laser

**Figure 4:**
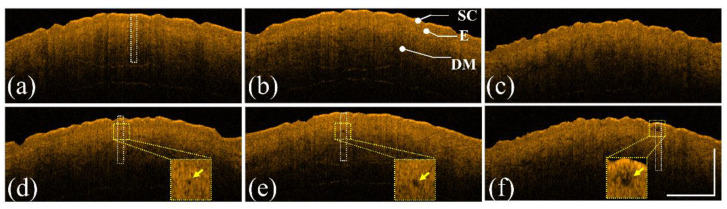
(**a**–**c**) 2D OCT images of healthy forearm skin of a 26-year-old female obtained before laser exposure. (**d**–**f**) OCT images corresponding to (**a**–**c**) after exposure to the fractional Nd:YAG laser with energies of 1.3, 1.9, and 2.5 mJ/microbeam, respectively. The regions indicated by the yellow squares are magnified and shown at the lower corners in (**d**–**f**), and the yellow arrows indicate the induced LIOBs. The scale bar represents 1 mm in length. SC: stratum corneum, E: epidermis, and DM: dermis.

**Figure 5:**
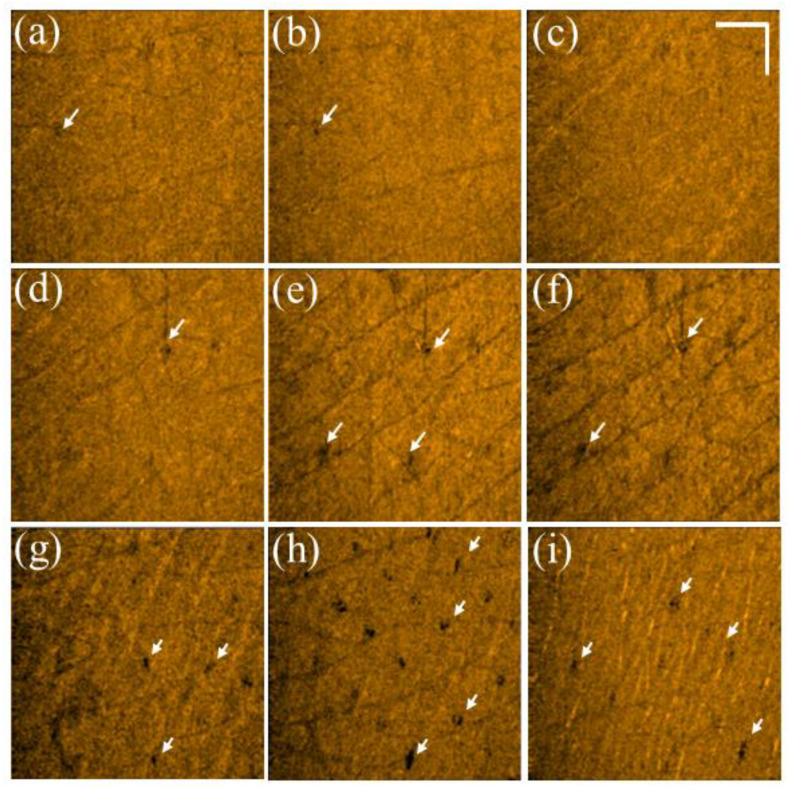
(**a**–**c**) En face images of skin after exposure to the fractional Nd:YAG laser with an energy of 1.3 mJ/microbeam obtained at depths of 300, 330, and 360 μm, respectively. (**d**–**f**) En face images of skin after laser exposure with an exposure energy of 1.9 mJ/microbeam obtained at depths of 430, 460, and 490 μm, respectively. (**g**–**i**) En face images of skin after laser exposure with an energy of 2.5 mJ/microbeam obtained at depths of 150, 180, and 210 μm, respectively. The white arrows indicate the induced vacuoles, and the scale bar represents 1 mm in length.

The same experimental procedures were repeated using a picosecond Nd:YAG laser to create LIOBs. The forearm skin of a 26-year-old female volunteer was exposed to a picosecond laser with exposure energies of 1.3, 1.9, and 2.5 mJ/microbeam. Figure 4a–c show the 2D OCT results of the forearm skin obtained before laser exposure and Figure 4d–f represent the 2D OCT images obtained after laser exposure with the abovementioned energies, respectively. The stratum corneum, epidermis, and dermis layers can be identified from the OCT results. The regions indicated by the yellow squares in Figure 4d–f show the induced LIOBs, and magnified images are shown at the lower corners in Figure 4d–f. Here, the yellow arrows indicate that the dark holes (vacuoles) are the induced LIOBs, which can be identified from the OCT results, showing that no backscattered intensity can be detected from the inner vacuoles. Additionally, it can be noted that the size of the vacuoles increased with the exposure energy. In addition to investigating the photodamage using 2D OCT images, the corresponding en face images are shown in Figure 5. Figure 5a–c show the en face images at depths of 300, 330, and 360 μm after exposure to 1.3 mJ/microbeam, and the white arrows indicate the induced vacuoles at different depths. Similarly, the en face results for exposure energy of 1.9 mJ/microbeam are shown in Figure 5d–f corresponding to the depths of 430, 460, and 490 μm, respectively. Figure 5g–i show the en face images at the depths of 150, 180, and 210 μm with an exposure energy of 2.5 mJ/microbeam. Note that the diameters of the vacuoles can be estimated from the OCT results.

### 3.3. Evaluation of the Attenuation Coefficient

Given that the optical properties of the skin, such as scattering and absorption, can be altered by photon energy, estimating the optical properties of the skin could be beneficial to evaluate the induced photodamage. Previous reports proposed various methods to assess the attenuation, scattering, and absorption coefficients from the OCT results [33,34,35]. Vermeer et al. proposed a depth-resolved method to estimate the attenuation coefficient of each pixel from OCT results [36]. Based on the proposed model, the OCT signal can be expressed as follows:

(1) $$I(z)=AL_{0}\mu (z)e^{-2\int_{0}^{z}\mu (i)di}$$

where *A* is a conversion factor, *μ*(*z*) is the depth-dependent attenuation coefficient, and *L*_0_ is the irradiance of the incident light beam on the tissue surface. Here, factor 2 accounts for one round trip of light propagation in tissue. Then, the integral of *I*(*z*) can be expressed as follows:

(2) $$\int I(z)dz=AL_{0}\int_{z}^{\infty }\mu (z)e^{-2\int_{0}^{z}\mu (i)di}dz=-\frac{I(z)}{2\mu (z)}+C$$

Then, as *I*(*z*) becomes zero when *z* is infinite, Equation (2) can be further simplified as follows:

(3) $$\int I(z)dz=\frac{I(z)}{2\mu (z)}$$

To estimate *μ*(*z*) in a limited depth range, *μ*(*z*) can be expressed as follows:

(4) $$\mu (z)=\frac{I(z)}{2\int_{z}^{\infty }I(u)du}≅\frac{I(z)}{2\int_{z}^{D}I(u)du}$$

where D is the selected depth for each A-scan. However, given that only discrete data can be obtained during the OCT measurements, Equation (4) can be revised as follows:

(5) $$\mu [n]\approx \frac{I[n]}{2\Delta z\cdot \sum_{n+1}^{n_{D}}I[n]}$$

where *n* is the nth pixel, *n_D_* is the pixel number of the selected depth, and Δ*z* represents the pixel size along the depth direction. Here, *μ*(*n*) is the averaged attenuation coefficient of the nth pixel.

**Figure 6:**
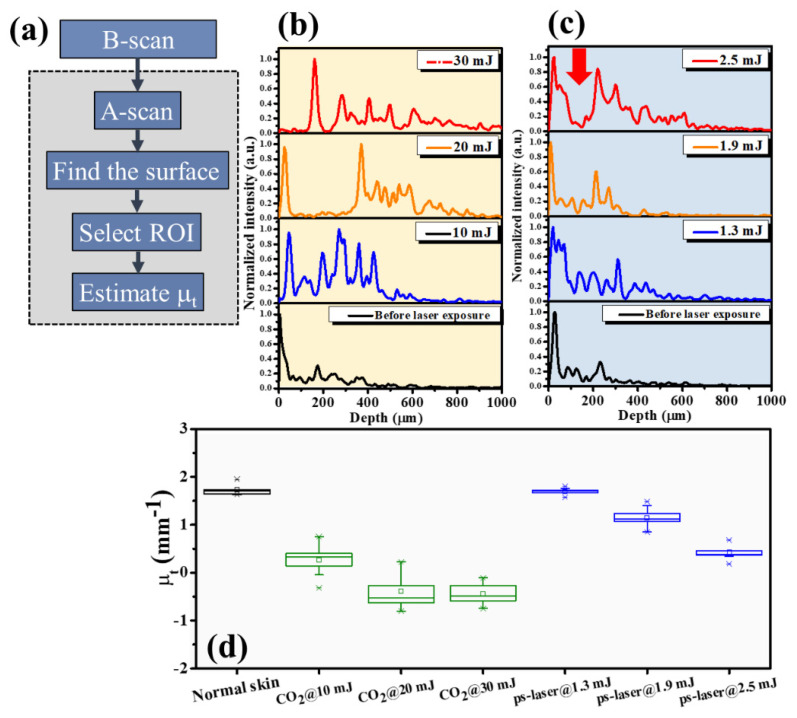
(**a**) Flowchart of the image processing for estimation of the attenuation coefficient. (**b**,**c**) Average A−scan profiles of the white rectangular regions in Figure 2 and Figure 4, respectively. (**d**) Statistical analysis of the attenuation coefficients estimated from normal skin, CO_2_ laser-exposed skin, and picosecond laser-exposed skin.

Thus, an image processing algorithm was developed to evaluate the attenuation coefficients of normal and laser-treated skin, whose flowchart is shown in Figure 6a. First, the skin surface was detected, and the attenuation coefficient of a selected depth range was estimated using Equation (5). Figure 6b shows the average A-scan profiles at four regions indicated by the white squares in Figure 2, corresponding to the intact skin structure before CO_2_ laser exposure and the exposed skin at 10, 20, and 30 mJ, respectively. Here, five adjacent A-scans were used to estimate the average A-scan profile. Figure 6b shows that the backscattered intensity varied with the photon energy. The surface peak disappeared after 30 mJ laser exposure due to tissue vaporization induced by the high exposure energy. Figure 6c plots the averaged A-scan profiles of skin locations indicated by the white rectangular squares in Figure 4. Figure 6c was obtained before exposure to the fractional picosecond laser and after laser exposure with 1.3, 1.9, and 2.5 mJ/microbeam. In contrast, no significant change in the backscattered intensity was observed when lower exposure energy was applied. However, higher exposure energy such as 2.5 mJ/microbeam caused a larger cavitation bubble, resulting in a significant change in the backscattered intensity, as indicated by the red arrow. Then, to compare the differences in the attenuation coefficients of normal skin, MAZ, and LIOB, eleven normal skin regions, eleven MAZs for each exposure energy level (10, 20, and 30, mJ/microbeams), and eleven LIOB locations for each energy level (1.3, 1.9, and 2.5 mJ/microbeam) were included for estimation of the attenuation coefficients. Figure 6d plots the results of the estimated attenuation coefficients. For normal skin, the average attenuation coefficient was 1.73 mm^−1^. The averaged attenuation coefficient decreased to −0.48 mm^−1^ after laser exposure at 30 mJ/microbeam. The negative attenuation coefficient for fractional CO_2_ laser exposure was due to the stronger backscattered stripes induced by the photon energy, as shown in Figure 2. Regarding the results for the fractional picosecond laser with an exposure energy of 1.3 mJ/microbeam, no significant change was observed in comparison to the estimated effects of normal skin without laser exposure. Still, the attenuation coefficients decreased with the exposure energy of the picosecond laser. The results indicate that the photodamage induced by the fractional CO_2_ and picosecond lasers can be identified from the estimated effects of the attenuation coefficient. Moreover, the photodamage along with the depth can be differentiated by the attenuation coefficient.

**Figure 7:**
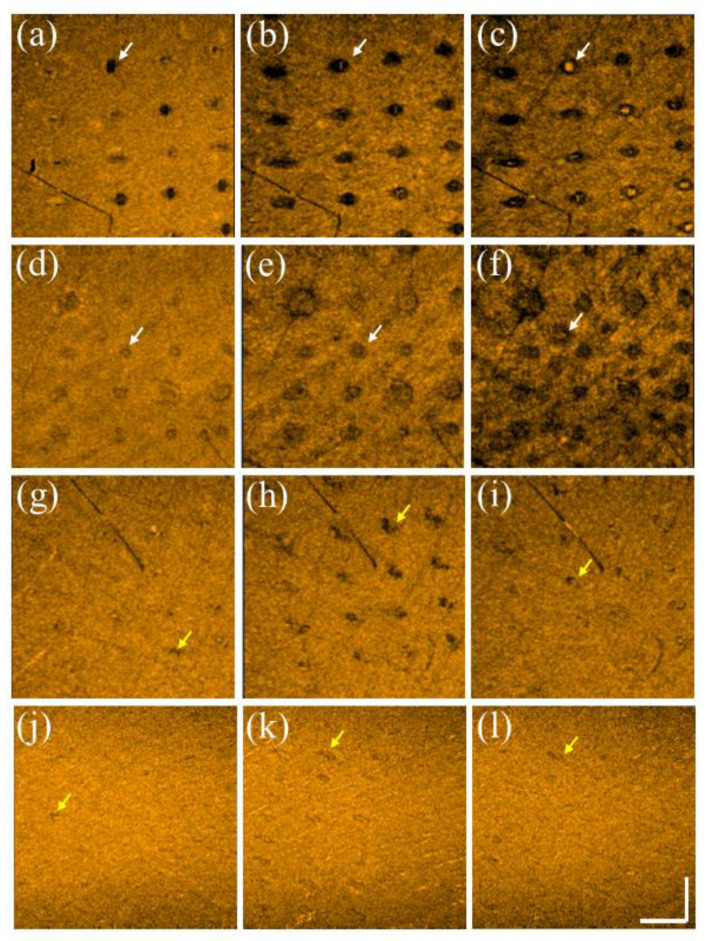
En face images were obtained at depths of 200, 400, and 600 μm on (**a**–**c**) Day 1 and (**d**–**f**) Day 7 after CO_2_ laser exposure with an energy of 30 mJ/microbeam. En face images were obtained at depths of 220, 270, and 320 μm on (**g**–**i**) Day 1 and (**j**–**l**) Day 3 after picosecond laser exposure with an energy of 2.5 mJ/microbeams. The scale bar in (**l**) represents 1 mm in length.

In addition to studying the photodamage induced by the two lasers, tissue recovery was investigated. Here, the same experimental procedures, including applying the fractional CO_2_ and picosecond lasers, were conducted on the volunteers’ skin. Figure 7a–c show the en face images obtained at depths of 200, 400, and 600 μm beneath the skin surface after fractional CO_2_ laser exposure with an energy of 30 mJ/microbeam. The results show that the tissue at the shallow depth was vaporized, causing hole structures. Instead, stronger backscattered spots in the center of the MAZs can be identified resulting from the damaged tissue accumulated at deeper depths. The white arrows indicate the induced MAZs at different depths. Moreover, the damaged area increased with the depth, as shown in Figure 7a–c. Subsequently, Figure 7d–f represent the corresponding en face images obtained at the same depths on Day 7. It was difficult to scan the same regions exactly on Days 1 and 7 for comparison, but the close areas were chosen for OCT scanning. After seven days, the wounds could still be identified, but the recovered progress could also be determined from the OCT results. Additionally, Figure 7g–i show the en face results of the picosecond laser exposure with an energy of 2.5 mJ/microbeam on Day 1, and Figure 7j–l represent the results obtained on Day 3. Compared with the fractional CO_2_ laser results, the intra-dermal cavities induced by the picosecond laser, as indicated by the yellow arrows present almost at the focusing depth, and the extra damage to the surrounding tissue along with the depth and transverse directions can be significantly reduced. After three days, significant tissue recovery could be identified, and only slight scars, indicated by the yellow arrows, could be found at the focusing depth.

**Figure 8:**
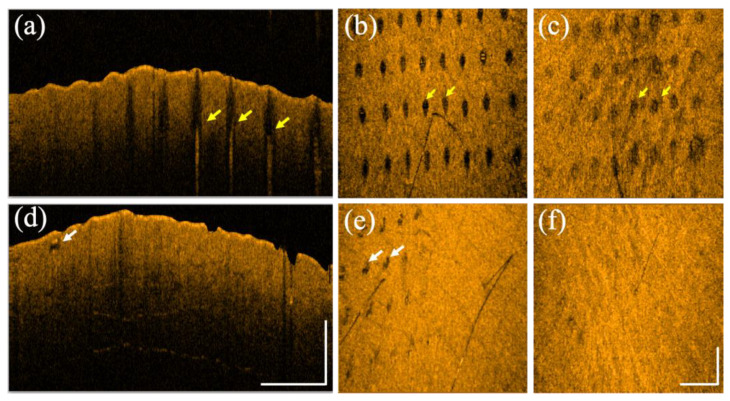
(**a**) 2D and (**b**) en face OCT images obtained from healthy forearm skin of a 28-year-old male on Day 1 after exposure to the fractional CO_2_ laser with an energy of 30 mJ/microbeam. (**c**) The corresponding en face image was obtained on Day 7 after exposure to the fractional CO_2_ laser. (**d**) 2D and (**e**) en face OCT images were obtained from healthy forearm skin of the same volunteer on Day 1 after exposure to the fractional Nd:YAG laser with an energy of 2.5 mJ/microbeam. (**f**) The corresponding en face image was obtained on Day 3 after exposure to the fractional Nd:YAG laser. The scale bars represent 1 mm in length.

Figure 8 shows the OCT scanning results of a 28-year-old male after exposure to both lasers on different sites of healthy forearm skin, respectively. Figure 8a,b represent the 2D and en face OCT images at a depth of 400 μm obtained on Day 1 after exposure to the fractional CO_2_ laser with an energy of 30 mJ/microbeam. Figure 8c shows the corresponding en face image at a depth of 400 μm obtained on Day 7 after exposure to the fractional CO_2_ laser. Figure 8d,e show the 2D and en face OCT images at a depth of 250 μm obtained on Day 1 after exposure to the fractional Nd:YAG laser with an energy of 2.50 mJ/microbeam. Figure 8f shows the corresponding en face image at a depth of 250 μm obtained on Day 3 after exposure to the fractional Nd:YAG laser. The yellow arrows indicate the MAZ structures, and the white arrows represent the induced vacuoles. Similarly, the induced vacuoles became unapparent after three days.

**Figure 9:**
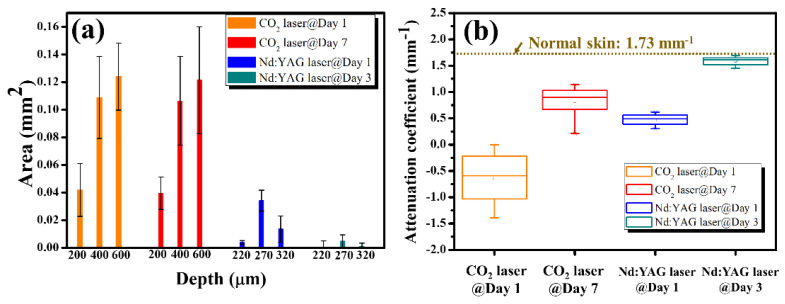
(**a**) Estimated damage areas obtained on Day 1 and Day 7 after CO_2_ laser treatment, and on Day 1 and Day 3 after exposure to the Nd:YAG laser. (**b**) Estimated attenuation coefficients obtained on Day 1 and Day 7 after CO_2_ laser treatment, and on Day 1 and Day 3 after exposure to the Nd:YAG laser.

Moreover, the damaged areas at various depths induced by both lasers can be estimated from the OCT images, and the attenuation coefficients can be compared during tissue recovery. The estimated damaged areas and the results of the attenuation coefficient are plotted in Figure 9a,b, respectively. The estimated effects of the fractional CO_2_ laser show that no significant improvement in the damaged areas at the three considered depths could be observed. In contrast, the damaged regions decreased rapidly on Day 3 for the fractional picosecond laser. Figure 9b shows that the estimated attenuation coefficient of the fractional CO_2_ laser treatment slightly decreased from Day 1 to Day 7 after treatment. In contrast to the fractional CO_2_ laser treatment results, the attenuation coefficient of the fractional Nd:YAG laser is close to that of the normal skin without laser treatment, which means that the tissue recovered in three days after exposure to the fractional picosecond Nd:YAG laser.

## 4. Discussion

In this study, we observed that the fractional Nd:YAG laser could cause a photoacoustic effect and other forms of intra-dermal cavities by the LIOB effect. The center wavelength of the CO_2_ laser is located at 10,600 nm, which can reach a deeper skin structure than the fractional Nd:YAG laser. The photon energy from the fractional CO_2_ laser can be absorbed by skin tissue and transferred to the thermal effect. The fractional CO_2_ laser enables ablation of the tissue surface and causes vertical photodamage ranging from the epidermis to the dermis layer. However, with a fractional Nd:YAG laser, the intradermal cavities only exist at a limited depth range that can be identified from the OCT results, and the tissue outside the depth range of LIOB can remain intact without photodamage.

With the fractionated picosecond laser, three energy levels were applied to cause cavitation bubbles with diameters ranging from tens to hundreds of micrometers. The results indicated that the diameter of the intradermal cavities increased with the exposure energy. Additionally, we observed that the existing depth of the induced cavity is not proportional to the exposure energy. On the contrary, the cavities were produced at a shallow depth when higher energy was applied, as shown in Figure 4, and the same phenomenon was also observed in histological examination [37,38,39]. The leading cause is that the photon energy can be easily absorbed by the plasma resulting from the LIOB effect, limiting the penetration depth of photons when a high energy level is implemented. However, because a low energy level does not achieve the threshold of LIOB out of the focus depth, the photons can reach deeper tissues.

To identify the photodamage and changes in the optical properties of the skin, we estimated the attenuation coefficients of normal and laser-treated skin. For normal skin tissue without laser exposure, the averaged attenuation coefficient of the forearm skin was calculated to be 1.73 mm^−1^. In contrast, the averaged attenuation coefficient decreased with the exposure energy when the skin was exposed to the fractional CO_2_ laser. However, there was no significant difference between the exposure energies of 20 and 30 mJ/microbeam, as shown in Figure 6, which means that both energy levels caused similar photodamage on tissue. The estimated attenuation coefficient became negative when exposed to higher energy, mainly resulting from the more robust backscattered strip induced by the photon energy, as shown in Figure 2.

Similarly, the attenuation coefficient decreased with increasing exposure energy when a fractional picosecond laser was used. When lower exposure energy of the picosecond laser was delivered, the induced cavitation bubble was relatively small, and no significant change in the backscattered intensity was observed, as shown in Figure 4 and Figure 6. Therefore, the average attenuation coefficient for 1.3 mJ exposure energy is close to the value of normal skin without laser exposure.

Given that the exposure of the fractional CO_2_ laser leads to striped damage from the epidermis to the dermis, a lengthy recovery period is required in comparison to the recovery time of the fractional picosecond laser. In this study, the wounds were repeatedly scanned after laser exposure, and 3D OCT images were recorded for comparison. As shown in Figure 9, the wounds at deeper depths in the experiment with the fractional CO_2_ laser did not change significantly on Day 7. Moreover, the estimated damaged area decreased at the shallow depths, as shown in Figure 7d. In contrast, the induced cavities after picosecond laser exposure became smaller on Day 3, and the upper and lower regions of the cavities became unapparent. The results demonstrate that the fractional picosecond laser causes less photodamage and reduces laser treatment recovery time.

## 5. Conclusions

We applied OCT for the non-invasive assessment of the photothermal and photoacoustic effects induced by fractional CO_2_ and Nd:YAG lasers, respectively. Furthermore, the OCT system was used to follow-up on the tissue recovery. The OCT results showed that the CO_2_ laser caused vertical damage from the skin surface to the dermal layer and the damaged area of the induced MAZ increased with the exposure energy. In contrast, the induced LIOBs by the fractionated Nd:YAG laser were produced at specific depths, and the size of the cavity increased with the exposure energy. To identify the change in the optical properties of the skin, we estimated the attenuation coefficients of normal and laser-treated skin. For normal skin tissue without laser exposure, the averaged attenuation coefficient of the forearm skin was calculated to be 1.73 mm^−1^, and the attenuation coefficient became smaller after laser exposure. Such attenuation coefficient estimation can be used to quantitatively evaluate photodamage and tissue recovery. In comparison, the recovery period of the fractional picosecond laser was shorter than that of the fractional CO_2_ laser. The results illustrate that the proposed method can be used to evaluate laser-induced tissue damage and recovery.

## Data Availability

No new data were created or analyzed in this study. Data sharing is not applicable to this article.
